# Thin single crystal perovskite solar cells to harvest below-bandgap light absorption

**DOI:** 10.1038/s41467-017-02039-5

**Published:** 2017-12-01

**Authors:** Zhaolai Chen, Qingfeng Dong, Ye Liu, Chunxiong Bao, Yanjun Fang, Yun Lin, Shi Tang, Qi Wang, Xun Xiao, Yang Bai, Yehao Deng, Jinsong Huang

**Affiliations:** 1 Department of Mechanical and Materials Engineering, University of Nebraska, Lincoln, NE 68588 USA; 2 Department of Applied Physical Sciences, University of North Carolina, Chapel Hill, NC 27599 USA

## Abstract

The efficiency of perovskite solar cells has surged in the past few years, while the bandgaps of current perovskite materials for record efficiencies are much larger than the optimal value, which makes the efficiency far lower than the Shockley–Queisser efficiency limit. Here we show that utilizing the below-bandgap absorption of perovskite single crystals can narrow down their effective optical bandgap without changing the composition. Thin methylammonium lead triiodide single crystals with tuned thickness of tens of micrometers are directly grown on hole-transport-layer covered substrates by a hydrophobic interface confined lateral crystal growth method. The spectral response of the methylammonium lead triiodide single crystal solar cells is extended to 820 nm, 20 nm broader than the corresponding polycrystalline thin-film solar cells. The open-circuit voltage and fill factor are not sacrificed, resulting in an efficiency of 17.8% for single crystal perovskite solar cells.

## Abstract (web summary)

Thin films of halide perovskites are promising for solar cell technology but they do not perform well at the band edge due to the low optical absorption. Herein, Chen et al. fabricate a high efficiency single crystal perovskite solar cell with thicker single crystals to harvest the below-bandgap photons.

## Introduction

Organic–inorganic halide perovskites (OIHPs) have attracted tremendous attentions for solar cell application in the past few years^1–9^ due to the superior optoelectronic properties, such as large extinction coefficient^10^, long carrier recombination lifetime^11^, large carrier mobility^12^, unique defect physics^13^, etc. The efficiency of OIHP solar cells has reached 22.1% quickly after development in a few years by the improvement of material quality and device interface^9^, but it is facing a bottleneck caused by the non-optimized bandgap of the current perovskite materials. The most widely studied perovskite solar cells are based on CH_3_NH_3_PbI_3_ (MAPbI_3_), which has a bandgap too large for single-junction solar cells^14^. To reduce the bandgap of MAPbI_3_, composition engineering has been developed by partially replacing MA^+^ and Pb^2+^ with FA^+^ and Sn^2+^, respectively^15–18^, which however brings new challenges. The easy oxidation of Sn^2+^ to Sn^4+^ causes additional severe chemical instability of the Sn-containing perovskite^17^. Introduction of excessive FA^+^ ions can cause the instability of material structure^16^.

Here we exploit an approach to broaden the absorption spectrum of perovskites by utilizing their below-bandgap transition. Below-bandgap absorption has been identified in MAPbI_3_ in previous studies, which was attributed to the indirect-bandgap absorption transition with a bandgap of ~60 meV smaller than the direct bandgap^19–22^. We found other perovskite materials, such as MAPbBr_3_, also showed below-gap absorption. Similar to silicon single crystal solar cells, further improvement of the device efficiency may be achieved by utilizing the below-bandgap absorption. However, the absorption coefficient corresponding to the below-bandgap transition is several orders magnitude smaller than that of the above-gap transition. Therefore, the thickness of the perovskite films thus needs to be over several micrometers (µm) or more, which is however beyond the carrier diffusion length in existing polycrystalline perovskite films. Perovskite single crystals are shown to have much longer carrier diffusion length well above tens of micrometer due to the absence of grain boundaries and significantly reduced defect density^23–26^. Current studies of the intrinsic properties of the perovskites are mainly based on thick bulk single crystals with thickness of millimeter^27,28^ which are too thick for application in solar cells. Growing thin perovskite single crystals with large area represents an effective approach to overcome this obstacle^29–31^, however there is no effective method handling the micrometer-thick iodide-based perovskite single crystals with large area. Recently, Liu et al.^32^ reported an efficiency of about 4% for perovskite single crystal solar cells. The low efficiency was due to the too large thickness of 50 μm for the perovskite crystal and non-passivated surface defects.

Herein, we report the growth of perovskite single crystals directly onto hole transport layer (HTL) covered indium tin oxide (ITO) substrates with controlled thickness of tens of micrometer and area of tens of millimeter square (mm^2^). Solar cells based on the thin single crystals show obviously broader spectral response compared to the polycrystalline thin-film solar cells, while the open-circuit voltage (*V*_OC_) and fill factor (FF) of the solar cells remain comparable to those of polycrystalline thin-film solar cells, which demonstrates the potential for the application of perovskite single crystals to further boost the efficiency of perovskite solar cells.

## Results

### Thickness-dependent device performance

**Fig. 1:**
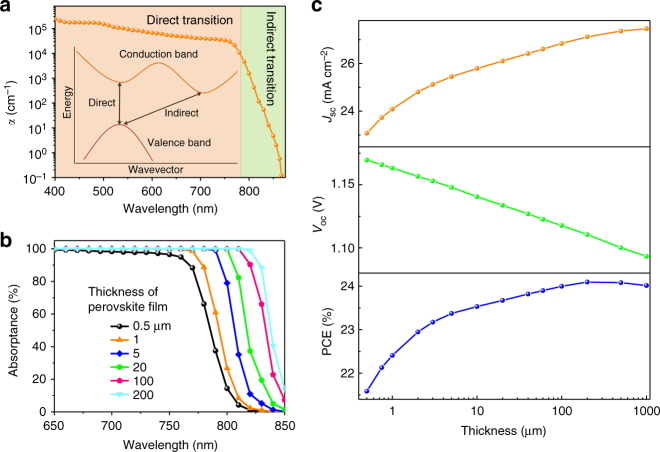
Thickness-dependent perovskite absorption and device performance. **a** Schematic illustration of the direct, below-bandgap transitions and absorption coefficient of methylammonium lead triiodide (MAPbI_3_) from a polycrystalline film. **b** The calculated absorption of MAPbI_3_ films with different thickness. **c** The calculated ideal dependence of *J*_sc_, *V*_oc_, and PCE of the single crystal solar cells on the thickness of the thin single crystals

We first simulate the thickness-dependent absorption of MAPbI_3_ and device efficiency to find out the feasibility of increasing device efficiency by utilizing the below-bandgap transition in MAPbI_3_ and the details are shown in the “Method” section. Figure 1a shows an absorption coefficient curve of MAPbI_3_ from literature^33^, which does show two–four orders magnitude weaker absorption for below-bandgap transition than direct transition. Figure 1b shows the calculated thickness-dependent percentage of light absorbed by the MAPbI_3_ films in devices where a full reflection of the light by a metal back electrode is assumed. When the thickness of MAPbI_3_ films increases from 500 nm to 200 µm, the absorption spectrum expands significantly to near-infrared region, leading to the red-shift of the absorption edge to 850 nm. The enhanced absorption in this wavelength range is important to increase the short-circuit current density (*J*_SC_), because sunlight has a significant portion of energy in this wavelength range. As shown in Fig. 1c, as the thickness of perovskite crystals increases from 500 nm to 200 µm, (*J*_SC_) increases from 23.0 mA cm^−2^ to 27.1 mA cm^−2^ due to the broadened absorption spectrum. The *V*_OC_ of the devices, however, has an opposite variation trend with respect to the thickness of absorber based on the diode equation:

1 $$V_{\rm{OC}\,} = \frac{{kT}}{q}{\mathrm{In}}\left( {\frac{{J_{\rm{SC}}}}{{J_0}}} \right)$$

where *T*, *k*, *q*, and, *J*_0_ are the absolute temperature, Boltzmann constant, elementary charge, and reverse bias saturated dark current density, respectively. The details for the simulation of crystal thickness-dependent efficiency limit of the single crystal solar cells can be found in the Supplementary Note. Though increased *J*_SC_ slightly increases *V*_OC_, larger thickness of perovskite films increases the charge recombination during their transport and thus *J*_0_, which reduces *V*_OC_. The charge recombination dominates the variation of *V*_OC_ with a clear reduction with increasing crystal thickness, as shown in Fig. 1c. There should be an optimal absorber thickness that the perovskite single crystal solar cells reach the highest efficiency. To determine the optimal thickness of perovskite single crystals, we assumed a constant FF of 80%, 90% external quantum efficiency (EQE) over the whole absorption spectrum, which are all reasonable and are justified in the “Method” section. The power conversion efficiency (PCE) increases first and then decreases with increasing the thickness of single crystals, resulting in an optimal crystal thickness of 200 μm. When the crystal thickness is over 10 μm, the PCE is higher than 23.5%, which is far beyond the state-of-the-art values of MAPbI_3_ polycrystalline thin-film solar cells.

### Growth and characterization of thin crystals

**Fig. 2:**
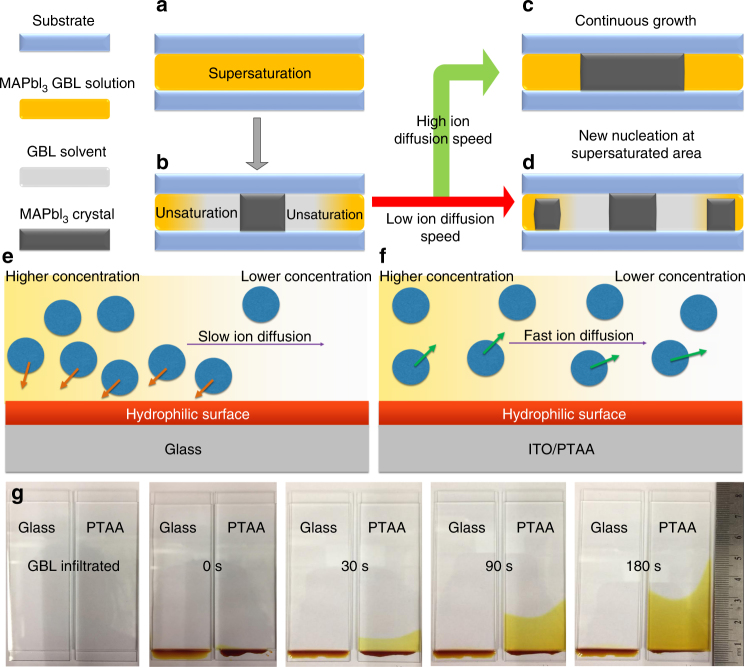
Growth mechanism of perovskite thin single crystals. **a**–**d** Schematic illustrations of correlation between ion diffusion and thin single-crystal growth. Schematic illustrations of ion diffusion rate in the confined gaps using **e** hydrophilic and **f** hydrophobic substrates. **g** Photographs of the diffusion process of methylammonium lead triiodide (MAPbI_3_) precursor solution in the confined gaps after different durations using hydrophilic glass and hydrophobic PTAA-covered ITO substrates

A diffusion-facilitated space-confined method was developed to obtain perovskite single crystals with controllable thickness down to micrometer scale. The confined space was constructed by two substrates (Fig. 2a), and the supersaturation of precursor solution was generated by the inverse solubility method^23^. The space-confined method was reported to grow thin perovskite singe crystals^31^, however, the crystal thickness could hardly go down to 100 µm for large-area perovskite crystals. The lateral crystal growth in this method can be limited by the inefficient long-range transportation of precursor ions along micrometer-size gap to continuously replenish the depleted precursors by crystal growth, which results in the formation of many small crystals or even polycrystals, as illustrated in Fig. 2d. When the gap is down to micrometer scale, the interaction between substrate surface and solvent in precursor solution becomes critically important for ion diffusion in solvent. Since the perovskite precursor ions generally form complex with the solvent^34^, the diffusion rate of precursor ions is determined by how fast the solvent molecules can transport on the substrate, and the surface tension determines how fast the solvent molecules close to the substrate can move along in-plane direction^35–37^. On wetting substrates, such as glass or ITO, the large surface tension imposes a friction force that drags down the ion diffusion^38^, as illustrated in Fig. 2e. Interestingly, when the substrates are covered with hydrophobic materials, such as PTAA^39^, which is also a hole transport material for perovskite solar cells, the ion diffusion is dramatically accelerated. The dramatically enhanced ion diffusion rate in micrometer-size gap enables a continuous growth of thin perovskite single crystals up to several millimeters along in-plane direction, as illustrated in Fig. 2c. To illustrate the dramatically enhanced ion diffusion rate by the surface modification, a drop of MAPbI_3_ precursor solution was placed at the end of two regular glass substrates (left in Fig. 2g) and two ITO substrates covered by PTAA (right in Fig. 2g), which had a same gap of 10 μm filled with pure γ-butyrolactone (GBL) solvent, and they were hold at the temperature for crystal growth (110 °C) or room temperature. Driven by the concentration gradient, the precursor ions diffused in the GBL solution from the bottom side to the topside, and the diffusion of ions could be visualized by the color change of the solution with diffusion rate estimated by motion of front edge at the interface of pure GBL and perovskite precursor solution. As expected, the edge propagation was very slow (0.01 mm s^−1^) in the gap of two glass substrates, while was at least 100-fold faster in the gap of two PTAA-covered ITO substrates (see Supplementary Video 1). We speculate that the much faster ion diffusion can be ascribed to the excellent lubrication effect of the nonwetting PTAA surface^38^. The nonwetting surface weakens the solvent–substrate interaction at the substrate surface, which will promote the diffusion of solvent. Therefore, the ions that form complexes with GBL molecules would not be dragged during the diffusion, as illustrated in Fig. 2f.

**Fig. 3:**
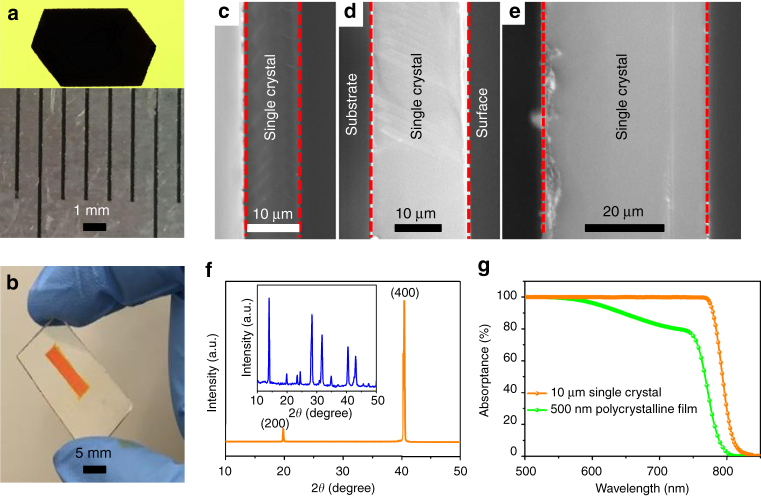
Characterization of perovskite thin single crystals. Photographs of **a** methylammonium lead triiodide (MAPbI_3_) thin single crystal and **b** methylammonium lead tribromide (MAPbBr_3_) thin single crystal using the hydrophobic interface confined lateral growth method. Cross-sectional SEM images of the MAPbI_3_ thin single crystals with different thickness: **c** ≈10 μm, **d** ≈20 μm, **e** ≈40 μm. **f** X-ray diffraction patterns of a MAPbI_3_ thin single crystal and the grounded powder (inset). **g** Absorption spectra of a 10-μm-thick MAPbI_3_ thin single crystal and a 500-nm-thick polycrystalline film

**Fig. 4:**
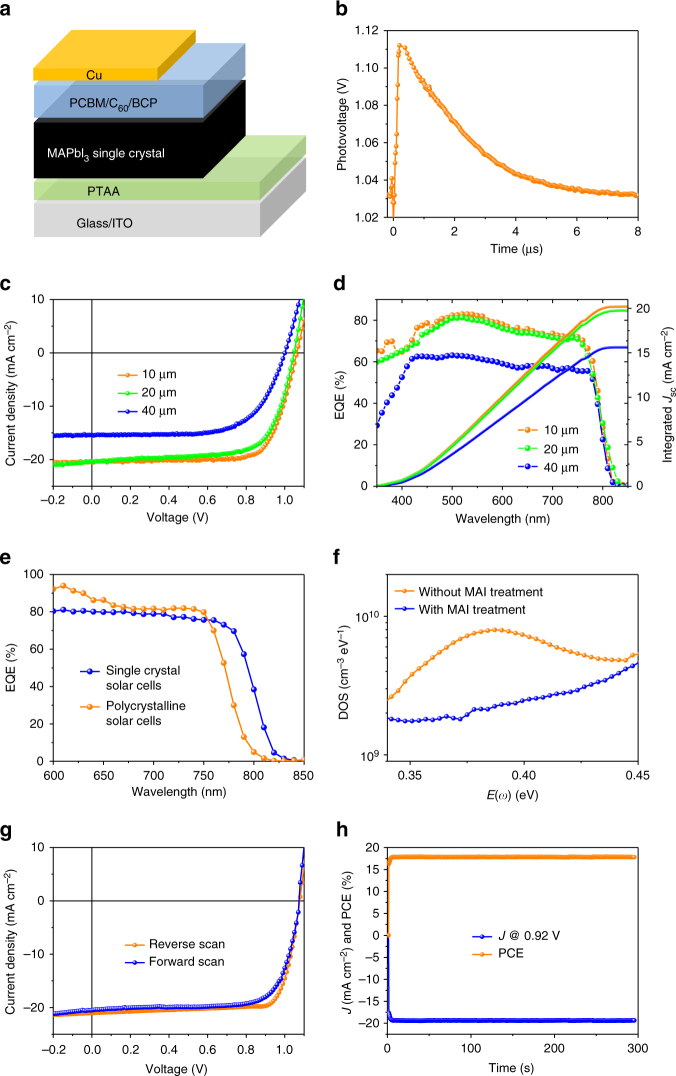
Characterization of perovskite single-crystal solar cells. **a** Device structure of the single-crystal solar cells. **b** Transient photovoltaic curve of a single-crystal solar cell under one-sun illumination. **c** Current density–voltage and **d** external quantum efficiency (EQE) curves and integrated current density of the optimal single-crystal solar cells using methylammonium lead triiodide thin single crystal with different thickness. **e** EQE of single-crystal solar cells and polycrystalline thin-film solar cells. **f** Trap density of states of the single-crystal solar cells before and after methylammonium iodide (MAI) treatment. **g** Current density–voltage curves of the single-crystal solar cells after MAI treatment measured under different scanning directions. **h** Stabilized *J*_sc_ and PCE of single-crystal solar cells with MAI treatment. The thicknesses of the single crystals in Fig. **e**–**h** and polycrystalline thin film are 10 and 500 nm, respectively

The fast ion transportation in the gap between two PTAA-covered substrates enables continuous growth of thin perovskite single crystals to a large area. The thin single crystals were grown on a PTAA-covered ITO substrate and the other PTAA-covered ITO substrate could be easily peeled off. The method is universally applicable to both iodide- and bromide-based perovskites. Figure 3a, b shows the photographs of as-grown millimeter-size MAPbI_3_ and MAPbBr_3_ single crystals with thickness of 10–20 μm. As shown in Fig. 3c–e, thin MAPbI_3_ single crystals are obtained with tuned thickness from 10 to 40 μm. For single crystals with thickness smaller than 10 μm, small-area multicrystals with lots of cracks were usually formed, which led to poor PCE for solar cells. The X-ray diffraction (XRD) pattern of a thin MAPbI_3_ single crystal in Fig. 3f shows only two sharp diffraction peaks which can be assigned to (200) and (400) planes, indicating that the crystal is (100) oriented. The corresponding rocking curve displays a small full-width at half-maximum of 0.33° for the (400) plane (Supplementary Fig. 1a), confirming the good quality of the thin single crystal. The 2D XRD shown in Supplementary Fig. 1b consists of scattered diffraction spots rather than a continuous arc, proving the single-crystal character of the grown materials. XRD pattern of the powders scratched from the thin single crystals (inset of Fig. 3f) confirms the tetragonal structure of these crystals at room temperature. The top-view SEM image of the thin single crystal (Supplementary Fig. 2) shows that the surface of the thin single crystal is smooth. Figure 3g shows the single-path absorption spectra of a single crystal with thickness of 10 μm and a polycrystalline thin film with thickness of 500 nm, which manifests an obvious redshift of the absorption spectrum edge from 790 to 810 nm. The redshift of the absorption spectrum is general for other kinds of perovskites, such as MAPbBr_3_, Cs_0.05_(FA_0.85_MA_0.15_)_0.95_Pb(I_0.85_Br_0.15_)_3_ (Supplementary Fig. 3) and FAPbI_3_^24^. The hole and electron mobility of the thin single crystals measured by the space charge limit current (SCLC) method (Supplementary Fig. 4) are 121 ± 15 cm^2^ V^−1^ s^−1^ and 36.8 ± 3.7 cm^2^ V^−1^ s^−1^, which are comparable to the values of bulk single crystals, indicating the good quality of the as-grown thin single crystals. The carrier recombination lifetime was measured by the transient photovoltaic (TPV) measurement under one-sun illumination (Fig. 4b). The obtained carrier lifetime is 2.2 ± 0.5 μs, which is shorter than that of bulk single crystals measured by the same method, because the smaller volume of the thin single crystals increases the carrier density, thus increasing the bimolecular recombination. In addition, carriers in the thinner single crystals are more susceptible to surface recombination by a quicker transit to the crystal surface. Combining the measured mobility and lifetime, the hole diffusion length and electron diffusion length under one-sun light are calculated to be 24.7 and 13.6 μm, respectively.

### Perovskite single-crystal solar cells

Single-crystal solar cells with device structure of ITO/PTAA/MAPbI_3_/phenyl-C_61_-butyric acid methyl ester (PCBM)/C_60_/bathocuproine (BCP)/copper (Cu) (Fig. 4a) were fabricated by depositing electron transport layers (ETLs) and metal electrodes on the top of the single crystals with the easy handling of ITO substrates. Figure 4c, d shows the thickness-dependent J–V and EQE curves of the single-crystal solar cells. The highest device efficiency is obtained when using single crystals with thickness of 10 μm, which is consistent with the measured carrier diffusion length. The best device has a *J*_SC_ of 20.5 mA cm^−2^, a *V*_OC_ of 1.06 V, a FF of 74.1%, and a PCE of 16.1%. As shown in Fig. 4e, the EQE spectrum of this device shows an EQE cutoff at 820 nm, corresponding to 20-nm redshift compared to that of the polycrystalline thin-film devices. The larger redshift of EQE spectrum than the absorption spectrum should be attributed to the reflection of light by the electrode, which doubles the effective crystal thickness for light absorbing. The statistics of PCE, FF, *J*_SC_, and *V*_OC_ for devices using single crystals with thickness of 10 μm are shown in Supplementary Fig. 6.

It is noted that thin single crystals may still have large surface charge traps^40^, though the bulk trap density should be very small. The surface charge traps are most likely generated from the loss of MAI when removing the single crystals out of the hot precursor solution^41^. Therefore, we treated the surface of the single crystals by spin coating MAI solution to reduce the surface charge trap density. This surface passivation effect was confirmed by the enhanced photoluminescence intensity (Supplementary Fig. 7). As shown in Fig. 4f, the surface trap density of the thin single crystals measured by thermal admittance spectroscopy is reduced by 2–5-folds after MAI treatment. The PCE of the best device also increases to 17.8% with a *J*_SC_ of 21.0 mA cm^−2^, a *V*_OC_ of 1.08 V, and a FF of 78.6% (Fig. 4g). The PCE of the device is confirmed by measuring the steady-state photocurrent output at the maximum power point (Fig. 4h). The increase of *J*_SC_ is consistent with the increase of the EQE values (Supplementary Fig. 8), and matches with the calculated *J*_SC_ from EQE. The corresponding statistics of PCE, FF, *J*_SC_, and *V*_OC_ are shown in Supplementary Fig. 9. By comparing with the calculated results in Fig. 1c, the *V*_OC_ is close to its predicted value of 1.14 V, and the FF is close to what is observed in high-efficiency polycrystalline perovskite solar cells. The main limiting parameter of the present single-crystal solar cells is the smaller *J*_SC_ than the predicted value of 25.8 mA cm^−2^, which may be caused by the enhanced light reflection on the much flatter single-crystal surface (Supplementary Fig. 10a), in addition to charge collection loss caused by the incompletely passivated surface defects on both surfaces of the single crystals (Supplementary Fig. 10b). Addressing these issues will have potential to further increase the efficiency.

Finally, we evaluate the stability of the single-crystal solar cells. We have demonstrated that bulk single crystals showed better moisture stability than the polycrystalline thin films due to the absence of grain boundaries^41^. Therefore, the air stability of the single-crystal solar cells should be better than that of polycrystalline thin-film solar cells. To confirm this, the shell lifetime of the single-crystal solar cells in air is investigated. As shown in Supplementary Fig. 11, the PCE shows almost no change after the device was stored in air under dark for 30 days, which is better than the polycrystalline thin-film solar cells.

### Conclusion

In conclusion, we demonstrated the use of a single crystal to broaden the photoresponse range of the perovskite solar cells without losing device photovoltage and fill factor. MAPbI_3_ thin single crystals were grown by a hydrophobic interface-confined lateral growth method, which yielded well control of crystal thickness at tens of micrometers. This work presents a new direction to further boost the efficiency and stability of perovskite solar cells. More efforts are needed to enhance the carrier diffusion length and passivate the surface nonradiative recombination center density in order to boost the device efficiency to SQ limit. The extended absorption can be attributed to the indirect bandgap absorption or Urbach band tail. Though there is some study that MAPbI_3_ has signature of indirect bandgap, there is no study whether the same conclusion can apply to MAPbBr_3_ or other perovskite materials yet. This study shows that these charges can actually be extracted out without being trapped.

## Methods

### Growth of perovskite thin single crystals

The perovskite thin single crystals were grown by a hydrophobic interface-confined lateral crystal growth method, as shown in Fig. 2. The PTAA film was prepared by spin coating 0.2 wt% PTAA solution in toluene at 4000 r.p.m. The as-prepared film was then thermally annealed at 100 °C for 10 min. As illustrated in Fig. 2a, 1.5 M MAPbI_3_ solution in GBL was inserted into two ITO/PTAA substrates, which were then placed on a hot plate with temperature of 100 °C for 1 h. Subsequently, the temperature was elevated to 110 and 120 °C to further promote the growth of the single crystals. Finally, the two substrates were separated to obtain the thin single crystals on ITO/PTAA substrates. The temperature was slowly decreased to room temperature (about 2 h) to ensure a good contact between PTAA and the single crystals.

### Device fabrication

The device fabrication was completed by depositing the electron transport layer and electrode. PCBM solution of 1 wt% in chlorobenzene was spun on top of the thin single crystal and then annealed at 50 °C for 10 min. After that, C60 (10 nm), BCP (3 nm), and Cu (80 nm) were thermally evaporated in sequential order. The C60 and BCP were evaporated on the whole area of the surface of the single crystal. The Cu electrode was evaporated with a mask by covering the edge of the single crystal to avoid shorts. For MAI treatment, 0.5 mg ml^−1^ MAI in IPA was spin coated on the single crystal before depositing PCBM and then annealed at 50 °C for 10 min.

### Characterization

The absorption spectra were recorded by using an Evolution 201 UV-Visible Spectrophotometer. First, the transmission spectra of the thin single crystal and polycrystalline thin film were measured. Subsequently, the corresponding absorption spectra were calculated as Absorptance = 1-Transmittance. The reflection spectrum was measured with the integrating sphere. The photocurrent density–voltage (J–V) curves of the devices were measured under AM 1.5-G irradiation (100 mW cm^−2^), which was produced by a xenon-lamp-based solar simulator (Oriel 67005, 150-W Solar Simulator). The light intensity was calibrated by a Schott visible-color glass-filtered (KG5 color-filtered) Si diode (Hamamatsu S1133) before photocurrent measurement. The J–V testing was performed with reverse scan direction at 0.1 V s^−1^ and sweep delay time was 10 ms. The EQE was obtained using a Newport QE measurement kit by focusing a monochromatic beam of light onto the devices. The X-ray diffraction patterns were obtained by a Rigaku D/Max-B X-ray diffractometer in the Bragg–Brentano parafocusing geometry. A conventional cobalt target X-ray tube equipped in the diffracted-beam monochromator was set to 40 kV and 30 mA. The SEM images were taken from a Quanta 200 FEG environmental scanning electron microscope. In the TPV measurement, the single-crystal solar cells were illuminated by laser pulses (337 nm, 4-ns width from a SRS nitrogen laser) from the ITO side to generate a transient photovoltage signal which functions as a small perturbation to the background open-circuit voltage. The transient voltage signal was recorded by a 1-GHz Agilent digital oscilloscope.

### Data availability

The data that support the findings of this study are available from the corresponding author on reasonable request.

## Electronic supplementary material

Supplementary Information
