# Supplementary material for: Thin single crystal perovskite solar cells to harvest below-bandgap light absorption
Source: Nat Commun. 2017 Dec 1;8:1890. doi: 10.1038/s41467-017-02039-5 (PMC5709415; doi:10.1038/s41467-017-02039-5)
Supplement: Supplementary file 1 — Supplementary Information [file 41467_2017_2039_MOESM1_ESM.pdf]

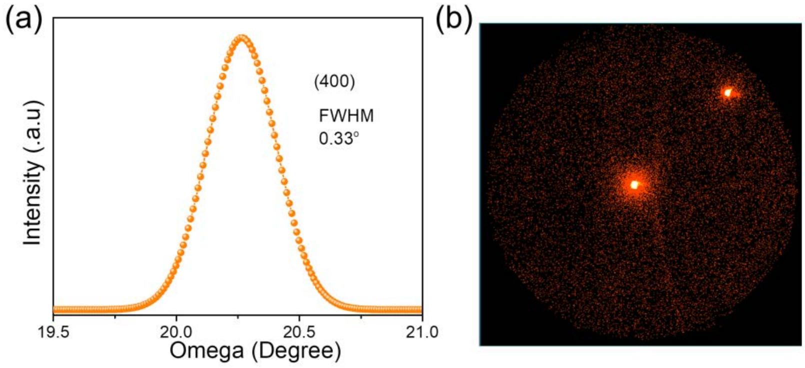

Supplementary Figure 1 (a) The rocking curve and (b) 2-D XRD of the thin single crystal with thickness of 40 μm. The indistinctive arc in the 2-D XRD is assigned to ITO.

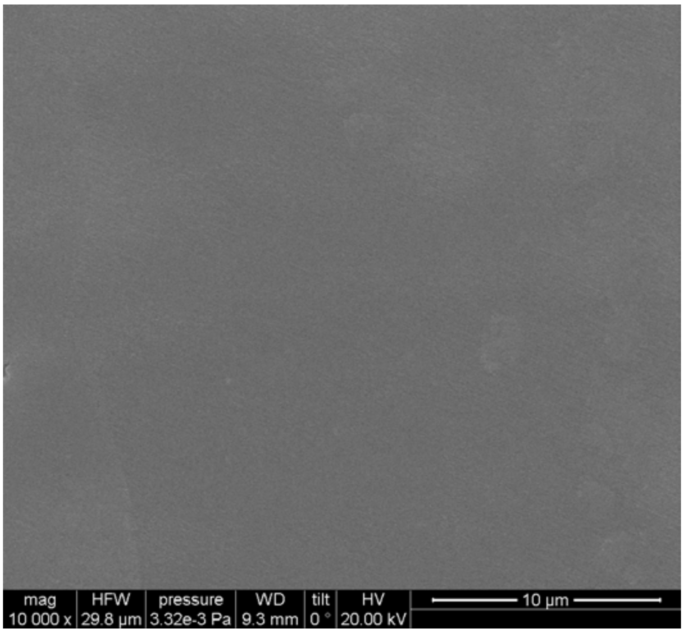

Supplementary Figure 2 Top-view SEM image of the thin single crystal with thickness of 10 μm.

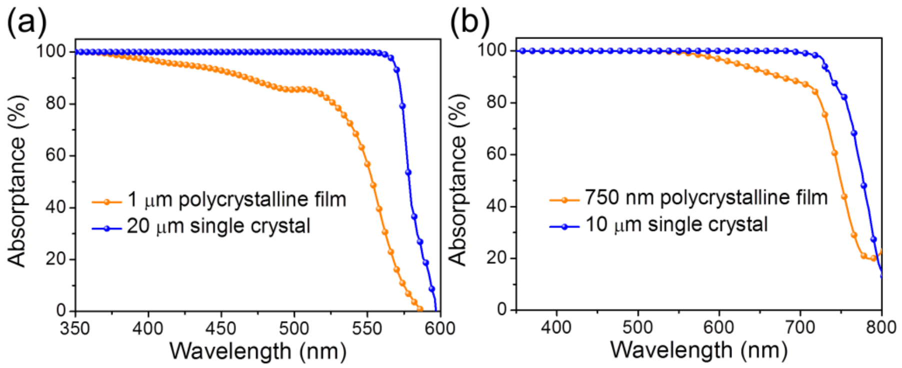

Supplementary Figure 3 Absorption spectra of the (a) methylammonium lead tribromide ($MAPbBr_3$) and (b) $Cs_{0.05}(FA_{0.85}MA_{0.15})_{0.95}Pb(I_{0.85}Br_{0.15})_3$ thin single crystals and polycrystalline films.

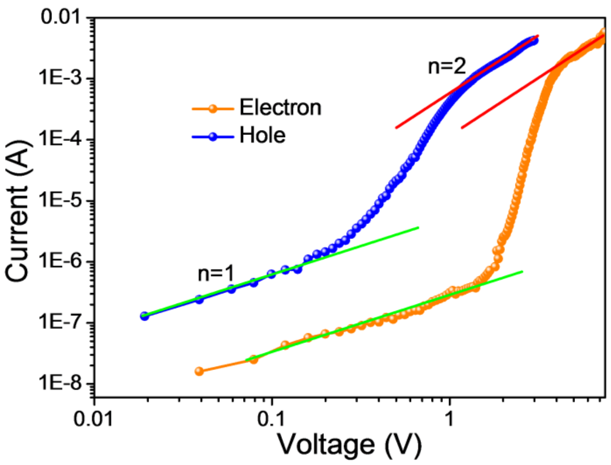

Supplementary Figure 4 Current-voltage curves of the hole-only and electron-only single crystal devices. The thickness of the single crystal is 10 μm.

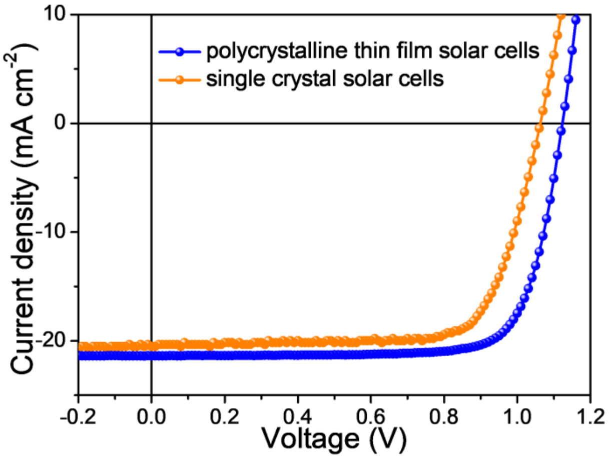

Supplementary Figure 5 J-V curves of single crystal solar cells with thickness of 10 μm and polycrystalline thin film solar cells with thickness of 500 nm.

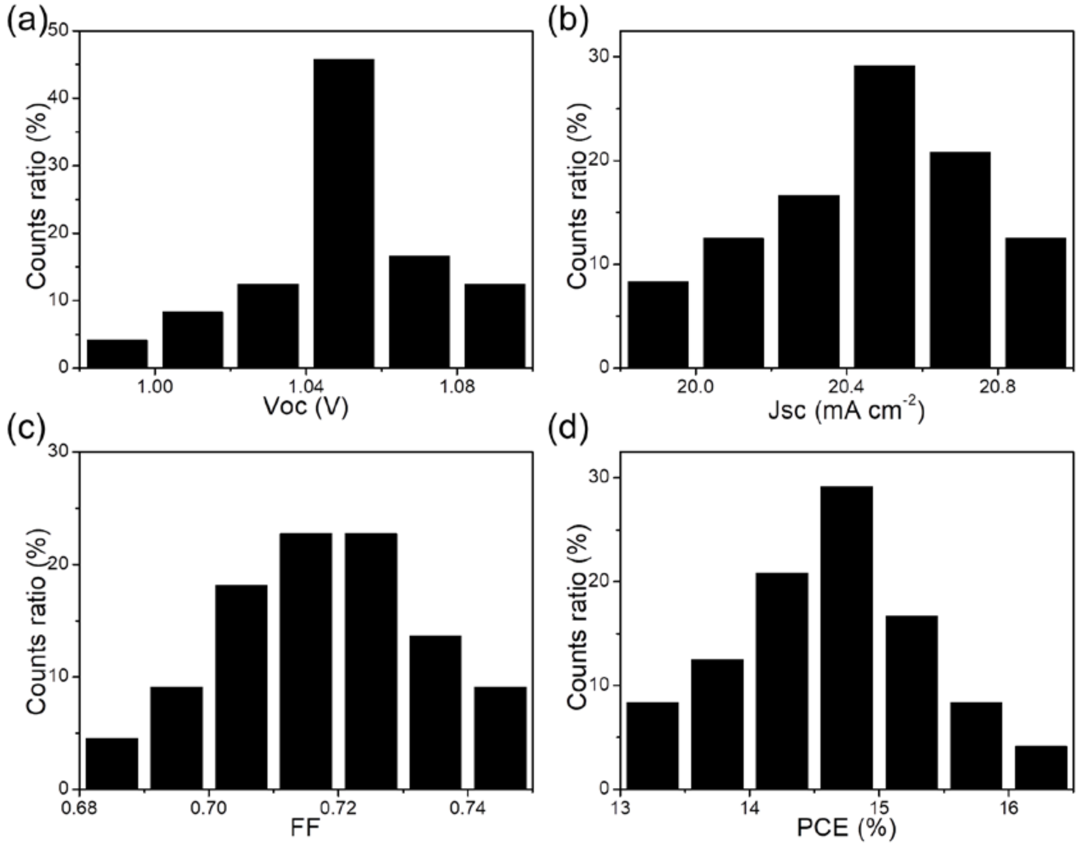

Supplementary Figure 6 Statistics of (a) *V*oc, (b) *J*sc, (c) FF, and (d) PCE distribution of the single crystal solar cells without MAI treatment. The thickness of the thin single crystal is 10 μm.

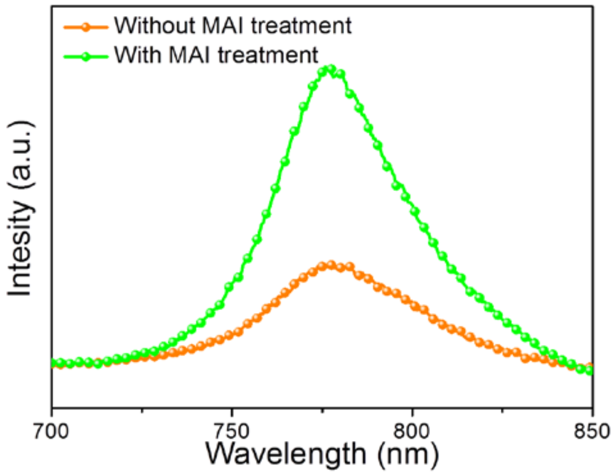

Supplementary Figure 7 Photoluminescence spectra of a methylammonium lead triiodide ($MAPbI_3$) single crystal before and after methylammonium iodide (MAI) treatment. The thickness of the thin single crystal is 10 μm.

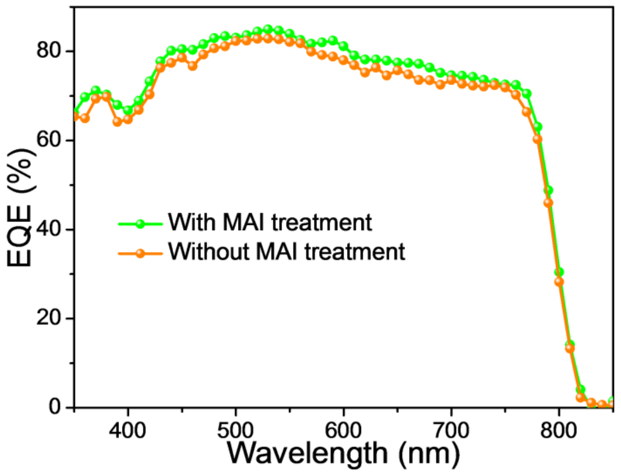

Supplementary Figure 8 EQE curves of the single crystal solar cells before and after methylammonium iodide (MAI) treatment. The thickness of the thin single crystal is 10 μm.

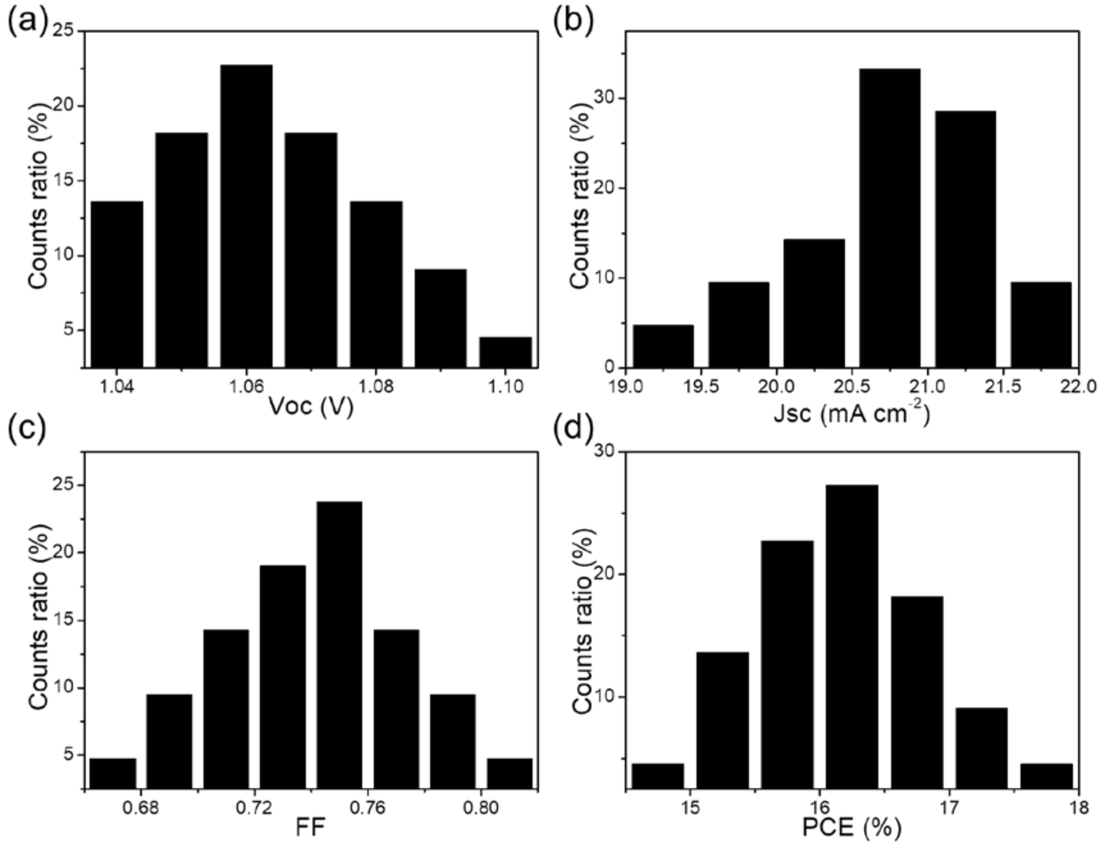

Supplementary Figure 9 Statistics of (a) *V*oc, (b) *J*sc, (c) FF, and (d) PCE distribution of the single crystal solar cells with methylammonium iodide (MAI) treatment. The thickness of the thin single crystal is 10 μm.

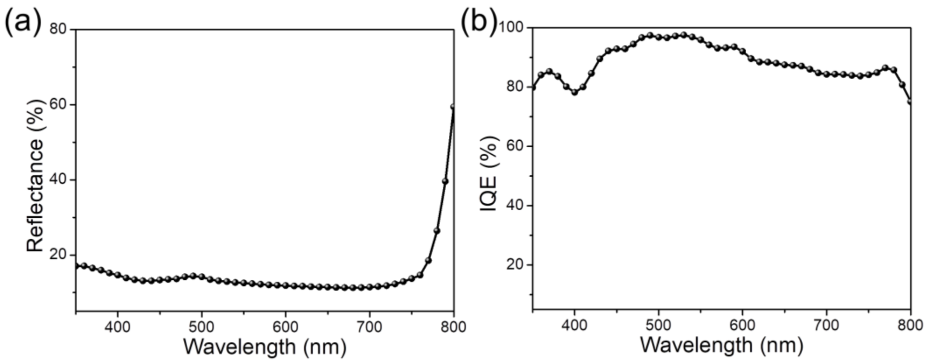

Supplementary Figure 10 (a) Reflection spectrum and (b) calculated internal quantum efficiency of the single crystal solar cells with thickness of 10 μm.

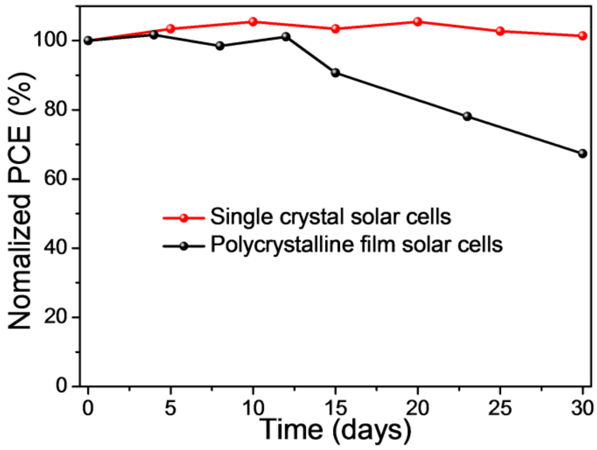

Supplementary Figure 11 Normalized PCE of the single crystal solar cells and polycrystalline film solar cells in ambient environment without encapsulation as a function of storage time. The temperature and relative humidity is 23 ℃and 30%.

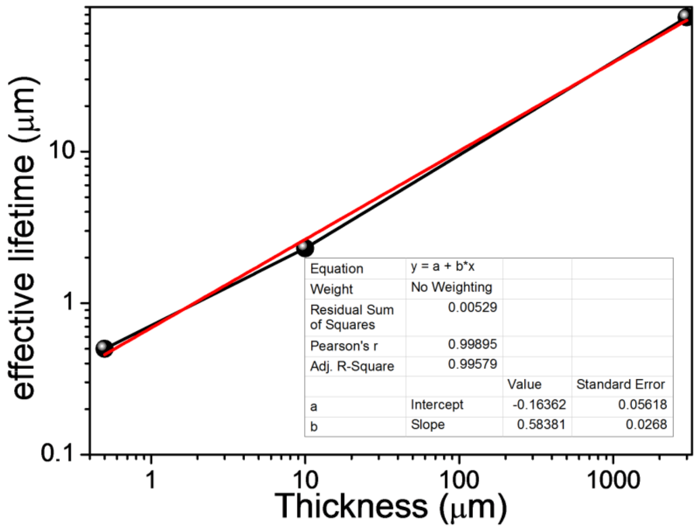

Supplementary Figure 12 Fitting curve of transient photovoltage values of methylammonium lead triiodide ($MAPbI_3$)polycrystalline film with thickness of 0.5 μm and single crystal with thickness of 10 μm and 3 mm.

Supplementary Notes. Calculation of the efficiency upper limit:

The J_{SC} was calculated by the integration of:

$$J_{SC} = q\int I(E)EQE(E)(1 - e^{-2\alpha(E)d})\, dE \quad (1)$$

where q is the electronic charge, I is the incident spectral photon flux density, α is the absorption coefficient, d is the thickness. A full reflection of light by the electron transport layer and the metal back electrode was assumed. The reflection of ITO glass was considered by assuming 90% EQE over the whole absorption spectrum which is reasonable because many high-efficiency perovskite solar cells demonstrated this high EQE. The bandgap of PTAA is large enough and its thickness is thin enough to avoid notable absorption, as demonstrated by the EQE of ~90% or above for the thin film polycrystalline perovskite solar cells with the same PTAA layer. Therefore, the parasitic absorption of PTAA was not considered. The UV-visible light is completely absorbed before approaching the PCBM/C_{60}/BCP layers due to the large absorption coefficient of perovskite in this wavelength region and large thickness of perovskite. The only portion of light that can partially approach the PCBM/C_{60}/BCP layers is almost at the band edge. But PCBM, C_{60} and BCP have negligible light absorption in the near-infrared region. Therefore, the light reflection was considered but parasitic absorption of these layers was not considered in the simulation.

The V_{OC} was calculated from the quasi-Fermi level splitting based on the nonequilibrium carrier concentration, resulting a relation of V_{OC} with J_{SC}, τ_{eff} and crystal thickness (d):

$$V_{OC} = \frac{kT}{q}\ln\left(\frac{J_{SC}N_{D}\tau_{eff}}{qn_{i}^{2}d}\right) \quad (2)$$

where T and k are the absolute temperature and Boltzmann constant, N_D is the concentration of the donor atom, τ_{eff} is effective recombination lifetime, n_i is the intrinsic carrier concentration, d is the film thickness. The τ_{eff} was obtained on measured effective lifetime and extracted lifetime at different $MAPbI_3$ film thickness (Supplementary Figure 12). The dependence of τ_{eff} on crystal thickness was derived based on:

$$\frac{1}{\tau_{\mathrm{eff}}} = \frac{1}{\tau_{\mathrm{s}}} + \frac{1}{\tau_{\mathrm{b}}} \quad (3)$$

$$\tau_{\mathrm{b}} = 1/(k_{\mathrm{b}} N_{\mathrm{D}} \frac{P}{Ed} \tau_{\mathrm{eff}}) \quad (4)$$

where τ_s and τ_b are surface recombination lifetime and bulk recombination lifetime, k_b is the bulk recombination rate constant, P is the irradiance of the incident light, E is the average energy of incident photons. The dependence of τ_{eff} on crystal thickness is derived to be:

$$\tau_{\mathrm{eff}} = \frac{-dE + \sqrt{d^2E^2 + 4k_{\mathrm{b}}N_{\mathrm{A}}PE\tau_{\mathrm{s}}^2 d}}{2k_{\mathrm{b}}N_{\mathrm{A}}I\tau_{\mathrm{s}}} \quad (5)$$

If the τ_s is much higher than the τ_b which is the case for perovskite single crystals, τ_{eff} is found to be proportional to the square root of the crystal thickness, which is consistent with the experimental results (Supplementary Figure 12). V_{OC} of 1.163V at thickness of 1 μm was obtained from the calculated result of Yin et al.[1]. Subsequently, the V_{OC} at other crystal thickness was calculated with reference to the 1 μm-thick device.

The FF was calculated by the empirical equation from single crystal silicon solar cells:

$$FF = \frac{qV\mathrm{oc}/kT - \ln(qV\mathrm{oc}/kT + 0.72)}{qV\mathrm{oc}/kT + 1} \quad (6)$$

The calculated FF overall remains almost unchanged with value of about 0.89 which is much larger than common values observed in high-efficiency perovskite solar cells. The FF was fixed at 0.8 when calculating the PCE. Combining the *J*sc, V_{OC} and FF, the dependence of PCE on film thickness was calculated.

Supplementary Method.

Measurement of carrier mobility and trap density by SCLC:

The hole-only devices were fabricated in a configuration of ITO/PTAA/$MAPbI_3$/Au. The hole mobility was calculated according to:

$$J_{\mathrm{D}} = \frac{9\varepsilon\varepsilon_0\mu V_{\mathrm{b}}^2}{8L^3} \tag{7}$$

where V_b is applied voltage. The calculated hole mobility is 121±15 $cm^2\ V^{-1}\ s^{-1}$. The hole trap density n_t in the thin single crystal was calculated according to:

$$V_{\mathrm{TFL}} = \frac{en_{\mathrm{t}}L^2}{2\varepsilon\varepsilon_0} \tag{8}$$

Where V_{TFL} is the voltage at which all the traps are filled, n_t is the hole trap density. The calculated hole trap density is $2.2\pm0.7\times10^{13}$ cm^{-3}. To measure the electron mobility, electron-only devices with configuration of ITO/PCBM/$MAPbI_3$/PCBM/C_{60}/BCP/Cu were fabricated. In this case, the thin single crystal was grown on ITO/PCBM substrate. The derived electron mobility and electron trap density are 36.8±3.7 $cm^2\ V^{-1}\ s^{-1}$ and $3.1\pm0.9\times10^{13}$ cm^{-3}.

Supplementary Reference

1. Yin, W.-J., Yang, J.-H., Kang, J., Yan, Y. & Wei, S.-H. Halide perovskite materials for solar cells: a theoretical review. *J. Mater. Chem. A* **3**, 8926-8942 (2015).
